# New provincial records of skinks (Squamata: Scincidae) from northwestern Vietnam

**DOI:** 10.3897/BDJ.3.e4284

**Published:** 2015-02-05

**Authors:** Anh Van Pham, Dzung Trung Le, Son Lan Hung Nguyen, Thomas Ziegler, Truong Quang Nguyen

**Affiliations:** † Faculty of Biology and Chemistry, Tay Bac University, Quyet Tam Ward, Son La City, Son La Province, Vietnam; ‡ Faculty of Biology, Hanoi National University of Education, 136 Xuan Thuy Street, Cau Giay, Hanoi, Vietnam; § AG Zoologischer Garten Köln, Riehler Straße 173, D-50735 Köln, Germany; | Institute of Ecology and Biological Resources, Vietnam Academy of Science and Technology, 18 Hoang Quoc Viet Road, Cau Giay, Hanoi, Vietnam

**Keywords:** Scincidae, new records, skinks, taxonomy, Dien Bien Province, Son La Province.

## Abstract

We report six new records of skinks from northwestern Vietnam: *Eutropis macularius, *Scincella devorator**, *S. monticola*, *S. ochracea*, *Sphenomorphus cryptotis* and *S. indicus*. Our new findings increase the species number of skinks (Scincidae) to nine in Dien Bien Province and to 14 in Son La Province. We also provide additional natural history data of aforementioned species.

## Introduction

In the recent checklist of the herpetofauna of Vietnam, Nguyen et al. (2009) listed 46 skink species of the family Scincidae. Since then five new species have been described from Vietnam, viz. *Scincella apraefrontalis* Nguyen, Nguyen, Böhme & Ziegler, 2010 (Nguyen et al. 2010b), *Scincella darevskii* Nguyen, Ananjeva, Orlov, Rybaltovsky & Böhme, 2010 (Nguyen et al. 2010d), *Tropidophorus boehmei* Nguyen, Nguyen, Schmitz, Orlov & Ziegler, 2010 (Nguyen et al. 2010e), *Sphenomorphus tonkinensis* Nguyen, Schmitz, Nguyen, Orlov, Böhme & Ziegler, 2011 (Nguyen et al. 2011), *Sphenomorphus sheai* Nguyen, Nguyen, Van Devender, Bonkowski & Ziegler, 2013 (Nguyen et al. 2013) and two species newly recorded for the country, namely *Sphenomorphus mimicus* Taylor, 1962 (Nguyen et al. 2011), and *Sphenomorphus incognitus* (Thompson, 1912) (Nguyen et al. 2012).

In Son La Province, previous studies documented a total of 11 species of Scincidae (Bobrov and Ho 1993, Nguyen et al. 2009, Nguyen et al. 2010). In Dien Bien Province, Do and Le (2009), Nguyen et al. (2009), and Nguyen et al. (2010d) recorded six species of skinks. During recent field work in northwestern Vietnam, a number of skinks was collected from Copia, Sop Cop and Muong Nhe nature reserves. Morphological examination revealed six new records of Scincidae for Son La and Dien Bien provinces that are provided herein.

## Materials and methods

### Sampling

**Figure 1.**
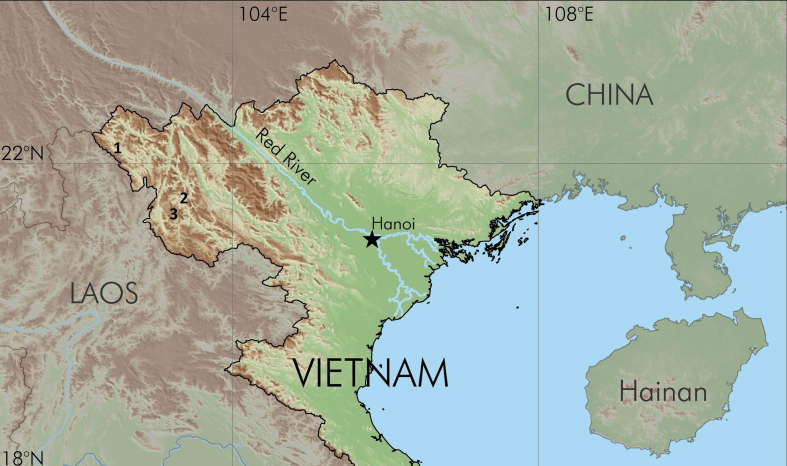
Map showing the survey sites: 1) Muong Nhe Nature Reserve in Dien Bien Province, 2) Copia Nature Reserve in Son La Province 3) Sop Cop Nature Reserve in Son La Provice, nothwestern Vietnam

Field surveys in northwestern Vietnam were conducted by Anh Van Pham, Tan Van Nguyen, Ngoc Thi Bich Nguyen, Men Thi Nguyen, and Hoang Van Tu (hereafter AVP et al.) between June 2013 and May 2014 and by Truong Quang Nguyen, Anh Van Pham, Hai Ngoc Ngo, Tan Van Nguyen, and Hoang Van Tu (hereafter TQN et al.) in September 2014 in the Copia and Sop Cop nature reserves (Son La Province); by Dzung Le Trung, Bach Viet Nguyen, Ngat Thi Bui, Hoa Phuong Vu (hereafter DTL et al.) between May 2012 and August 2014 in Muong Nhe Nature Reserve (Fig. 1). Specimens were collected by hand between 9:00–23:00. After taking photographs, specimens were anaesthetized in a closed vessel with a piece of cotton wool containing ethyl acetate, fixed in 80% ethanol and subsequently stored in 70% ethanol. Voucher specimens were subsequently deposited in the collections of the Tay Bac University (TBU), Son La Province and the Hanoi National University of Education (HNUE), Hanoi; and the Institute of Ecology and Biological Resources (IEBR), Hanoi, Vietnam.

### Morphological characters

Measurements were taken with a digital caliper to the nearest 0.1 mm. The following abbreviations are used: SVL: Snout-vent length, TaL: Tail length, AG: Axilla-groin distance (from posterior junction of forelimb and body wall to anterior junction of hind limb and body wall with the limbs held at right angles to the body), HL: Head length (from tip of snout to posterior margin of parietal), HW: Head width (at the widest point of temporal region), SL: Snout length (from anterior corner of eye to tip of snout), SFlL: Snout-forelimb length (from tip of snout to anterior junction of forelimb and body wall, with the limb held at right angles to the body), FlL: Forelimb length (from anterior junction of forelimb and body wall to the tip of fourth finger, with the limb held at right angles to the body), HlL: Hind limb length (anterior junction of hindlimb and body wall to the tip of fourth toe, with the limb held at right angles to the body). Scalation: Paravertebral scales (number of scales in a line from posterior edge of parietals to dorsal point opposite posterior margin of the medial precloacals); ventral scale rows (number of scales from first gular to anterior margin of precloacals).

## Taxon treatments

### Eutropis macularius

(Blyth, 1853)

#### Materials

- **Type status:** Other material. **Occurrence:** catalogNumber: TBU PAT.167; individualCount: 1; sex: male; lifeStage: adult; **Taxon:** scientificNameID: Eutropis macularius; scientificName: Eutropis macularius; class: Reptilia; order: Squamata; family: Scincidae; genus: Eutropis; specificEpithet: macularius; scientificNameAuthorship: (Blyth, 1853); **Location:** country: Vietnam; countryCode: VN; stateProvince: Son La; county: Song Ma; municipality: Huoi Mot; locality: Sop Cop Nature Reserve, near Pa Man Village; verbatimElevation: 500 m; verbatimLatitude: 21°02'27''N; verbatimLongitude: 103°41'40''E; verbatimCoordinateSystem: WGS84; **Event:** eventDate: May 1, 2014; eventRemarks: collected by AVP et al.; **Record Level:** language: en; collectionCode: Reptiles; basisOfRecord: PreservedSpecimen
- **Type status:** Other material. **Occurrence:** catalogNumber: TBU PAT.168; individualCount: 1; sex: male; lifeStage: adult; **Taxon:** scientificNameID: Eutropis macularius; scientificName: Eutropis macularius; class: Reptilia; order: Squamata; family: Scincidae; genus: Eutropis; specificEpithet: macularius; scientificNameAuthorship: (Blyth, 1853); **Location:** country: Vietnam; countryCode: VN; stateProvince: Son La; county: Song Ma; municipality: Huoi Mot; locality: Sop Cop Nature Reserve, near Pa Man Village; verbatimElevation: 500 m; verbatimLatitude: 21°02'27''N; verbatimLongitude: 103°41'40''E; verbatimCoordinateSystem: WGS84; **Event:** eventDate: May 1, 2014; eventRemarks: collected by AVP et al.; **Record Level:** language: en; collectionCode: Reptiles; basisOfRecord: PreservedSpecimen
- **Type status:** Other material. **Occurrence:** catalogNumber: TBU PAT.169; individualCount: 1; sex: male; lifeStage: adult; **Taxon:** scientificNameID: Eutropis macularius; scientificName: Eutropis macularius; class: Reptilia; order: Squamata; family: Scincidae; genus: Eutropis; specificEpithet: macularius; scientificNameAuthorship: (Blyth, 1853); **Location:** country: Vietnam; countryCode: VN; stateProvince: Son La; county: Song Ma; municipality: Huoi Mot; locality: Sop Cop Nature Reserve, near Pa Man Village; verbatimElevation: 500 m; verbatimLatitude: 21°02'27''N; verbatimLongitude: 103°41'40''E; verbatimCoordinateSystem: WGS84; **Event:** eventDate: May 1, 2014; eventRemarks: collected by AVP et al.; **Record Level:** language: en; collectionCode: Reptiles; basisOfRecord: PreservedSpecimen
- **Type status:** Other material. **Occurrence:** catalogNumber: TBU PAT.172; individualCount: 1; sex: male; lifeStage: adult; **Taxon:** scientificNameID: Eutropis macularius; scientificName: Eutropis macularius; class: Reptilia; order: Squamata; family: Scincidae; genus: Eutropis; specificEpithet: macularius; scientificNameAuthorship: (Blyth, 1853); **Location:** country: Vietnam; countryCode: VN; stateProvince: Son La; county: Song Ma; municipality: Huoi Mot; locality: Sop Cop Nature Reserve, near Pa Man Village; verbatimElevation: 500 m; verbatimLatitude: 21°02'27''N; verbatimLongitude: 103°41'40''E; verbatimCoordinateSystem: WGS84; **Event:** eventDate: May 1, 2014; eventRemarks: collected by AVP et al.; **Record Level:** language: en; collectionCode: Reptiles; basisOfRecord: PreservedSpecimen
- **Type status:** Other material. **Occurrence:** catalogNumber: TBU PAT.173; individualCount: 1; sex: male; lifeStage: adult; **Taxon:** scientificNameID: Eutropis macularius; scientificName: Eutropis macularius; class: Reptilia; order: Squamata; family: Scincidae; genus: Eutropis; specificEpithet: macularius; scientificNameAuthorship: (Blyth, 1853); **Location:** country: Vietnam; countryCode: VN; stateProvince: Son La; county: Song Ma; municipality: Huoi Mot; locality: Sop Cop Nature Reserve, near Pa Man Village; verbatimElevation: 500 m; verbatimLatitude: 21°02'27''N; verbatimLongitude: 103°41'40''E; verbatimCoordinateSystem: WGS84; **Event:** eventDate: May 1, 2014; eventRemarks: collected by AVP et al.; **Record Level:** language: en; collectionCode: Reptiles; basisOfRecord: PreservedSpecimen
- **Type status:** Other material. **Occurrence:** catalogNumber: TBU PAT.165; individualCount: 1; sex: female; lifeStage: adult; **Taxon:** scientificNameID: Eutropis macularius; scientificName: Eutropis macularius; class: Reptilia; order: Squamata; family: Scincidae; genus: Eutropis; specificEpithet: macularius; scientificNameAuthorship: (Blyth, 1853); **Location:** country: Vietnam; countryCode: VN; stateProvince: Son La; county: Song Ma; municipality: Huoi Mot; locality: Sop Cop Nature Reserve, near Pa Man Village; verbatimElevation: 500 m; verbatimLatitude: 21°02'27''N; verbatimLongitude: 103°41'40''E; verbatimCoordinateSystem: WGS84; **Event:** eventDate: May 1, 2014; eventRemarks: collected by AVP et al.; **Record Level:** language: en; collectionCode: Reptiles; basisOfRecord: PreservedSpecimen
- **Type status:** Other material. **Occurrence:** catalogNumber: TBU PAT.166; individualCount: 1; sex: female; lifeStage: adult; **Taxon:** scientificNameID: Eutropis macularius; scientificName: Eutropis macularius; class: Reptilia; order: Squamata; family: Scincidae; genus: Eutropis; specificEpithet: macularius; scientificNameAuthorship: (Blyth, 1853); **Location:** country: Vietnam; countryCode: VN; stateProvince: Son La; county: Song Ma; municipality: Huoi Mot; locality: Sop Cop Nature Reserve, near Pa Man Village; verbatimElevation: 500 m; verbatimLatitude: 21°02'27''N; verbatimLongitude: 103°41'40''E; verbatimCoordinateSystem: WGS84; **Event:** eventDate: May 1, 2014; eventRemarks: collected by AVP et al.; **Record Level:** language: en; collectionCode: Reptiles; basisOfRecord: PreservedSpecimen
- **Type status:** Other material. **Occurrence:** catalogNumber: TBU PAT.170; individualCount: 1; sex: female; lifeStage: adult; **Taxon:** scientificNameID: Eutropis macularius; scientificName: Eutropis macularius; class: Reptilia; order: Squamata; family: Scincidae; genus: Eutropis; specificEpithet: macularius; scientificNameAuthorship: (Blyth, 1853); **Location:** country: Vietnam; countryCode: VN; stateProvince: Son La; county: Song Ma; municipality: Huoi Mot; locality: Sop Cop Nature Reserve, near Pa Man Village; verbatimElevation: 500 m; verbatimLatitude: 21°02'27''N; verbatimLongitude: 103°41'40''E; verbatimCoordinateSystem: WGS84; **Event:** eventDate: May 1, 2014; eventRemarks: collected by AVP et al.; **Record Level:** language: en; collectionCode: Reptiles; basisOfRecord: PreservedSpecimen
- **Type status:** Other material. **Occurrence:** catalogNumber: TBU PAT.171; individualCount: 1; sex: female; lifeStage: adult; **Taxon:** scientificNameID: Eutropis macularius; scientificName: Eutropis macularius; class: Reptilia; order: Squamata; family: Scincidae; genus: Eutropis; specificEpithet: macularius; scientificNameAuthorship: (Blyth, 1853); **Location:** country: Vietnam; countryCode: VN; stateProvince: Son La; county: Song Ma; municipality: Huoi Mot; locality: Sop Cop Nature Reserve, near Pa Man Village; verbatimElevation: 500 m; verbatimLatitude: 21°02'27''N; verbatimLongitude: 103°41'40''E; verbatimCoordinateSystem: WGS84; **Event:** eventDate: May 1, 2014; eventRemarks: collected by AVP et al.; **Record Level:** language: en; collectionCode: Reptiles; basisOfRecord: PreservedSpecimen

#### Description

Morphological characters (determination after Smith 1935, Taylor 1963). Males: SVL 55.6–61.7 mm (mean ± SD 58.3 ± 2.4 mm, n = 5), TaL 95.4 mm (n = 1); females: SVL 55.4–59.6 mm (mean ± SD 57.5 ± 1.7 mm, n = 4), TaL 80.5–85.7 mm (mean ± SD 83.6 ± 2.7 mm, n = 3). For further measurements and proportions see Table 1.

Head longer than wide; rostral wider than high; supranasals present, separated from each other; prefrontals separated by frontal; parietals separated by interparietal; enlarged nuchal scales in one pair; loreals 2; supraciliaries 5 or 6; supraoculars 4, followed by 2 postsupraoculars; primary temporals 3, secondary temporals 3, keeled; supralabials 7, the fifth below the eye; external ear openings with small projecting lobules anteriorly, tympanum deeply sunk; mental wider than long; infralabials 7; postmental undivided; midbody scales in 30 rows; dorsal scales with 5– 7 obtuse keels, slightly larger than lateral scales; paravertebral scales 36–39; ventrals in 43–47 transverse rows, smooth; precloacals 2, enlarged; medial subcaudals not widened; limbs strong, pentadactyl; fingers and toes meeting when adpressed; subdigital lamellae smooth, 10 or 11 under fourth finger and 13–16 under fourth toe.

**Figure 2.**
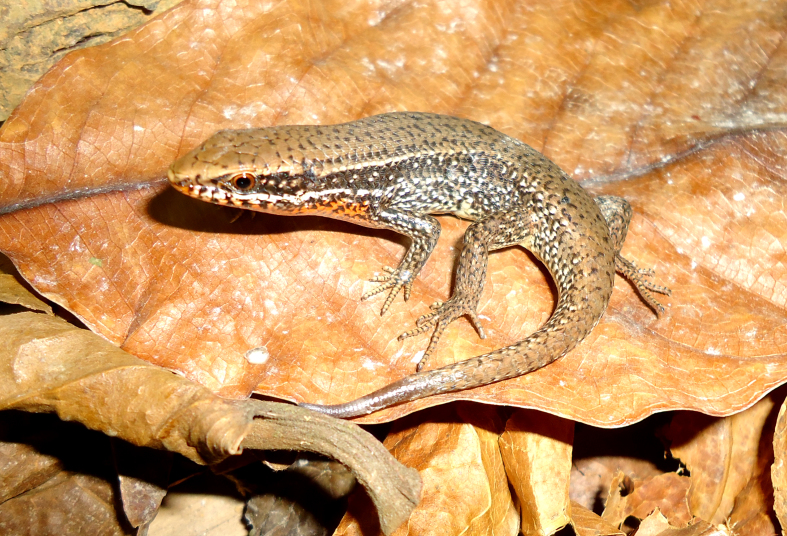
*Eutropis macularius* (TBU PAT.168, adult male) from Son La Province, Viet Nam

Coloration in alcohol. Dorsal surface brown, with or without small black spots; white stripe present on upper lip, extending backwards to shoulder; a dorsolateral light line extending from eye to midway on body; flank dark brown from behind the eye to hind limb, with white spots; neck and throat reddish in males and cream in females; venter and underside of tail cream. For coloration in life see Fig. 2.

#### Distribution

In Vietnam, this is a widespread species, known from Lang Son Province in the North to Kien Giang Province in the South. Elsewhere, *E. macularius* has been recorded from Pakistan, India, Bhutan, Sri Lanka, Bangladesh, Myanmar, Laos, Thailand, Cambodia, and Malaysia (Nguyen et al., 2009). This is a new record for Son La Province.

#### Ecology

Specimens of *E. macularius* were collected between 9:00 to 16:30 in the bamboo bush near the road. The surrounding habitat was disturbed secondary forest of small hardwood, bamboo and shrub.

### Scincella devorator

(Darevsky, Orlov & Ho, 2004)

#### Materials

- **Type status:** Other material. **Occurrence:** catalogNumber: TBU PAT.26; individualCount: 1; sex: female; lifeStage: adult; **Taxon:** scientificNameID: Scincella devorator; scientificName: Scincella devorator; class: Reptilia; order: Squamata; family: Scincidae; genus: Scincella; specificEpithet: devorator; scientificNameAuthorship: (Darevsky, Orlov & Ho, 2004); **Location:** country: Vietnam; countryCode: VN; stateProvince: Son La; county: Thuan Chau; municipality: Co Ma; locality: Copia Nature Reserve, near Hua Ty Village; verbatimElevation: 1590 m; verbatimLatitude: 21°19'43.8''N; verbatimLongitude: 103°35’6.6''E; verbatimCoordinateSystem: WGS84; **Event:** eventDate: June 12, 2013; eventRemarks: collected by AVP et al.; **Record Level:** language: en; collectionCode: Reptiles; basisOfRecord: PreservedSpecimen
- **Type status:** Other material. **Occurrence:** catalogNumber: TBU PAT.72; individualCount: 1; sex: female; lifeStage: adult; **Taxon:** scientificNameID: Scincella devorator; scientificName: Scincella devorator; class: Reptilia; order: Squamata; family: Scincidae; genus: Scincella; specificEpithet: devorator; scientificNameAuthorship: (Darevsky, Orlov & Ho, 2004); **Location:** country: Vietnam; countryCode: VN; stateProvince: Son La; county: Thuan Chau; municipality: Co Ma; locality: Copia Nature Reserve, near Hua Ty Village; verbatimElevation: 1540 m; verbatimLatitude: 21°20’12.2''N; verbatimLongitude: 103°34’49.7''E; verbatimCoordinateSystem: WGS84; **Event:** eventDate: August 2, 2013; eventRemarks: collected by AVP et al.; **Record Level:** language: en; collectionCode: Reptiles; basisOfRecord: PreservedSpecimen

#### Description

Morphological characters (determination after Darevsky et al. 2004). A medium-sized skink: SVL 51.2–53.9 mm (n = 2 females) TaL 65.7 mm (n = 1). For further measurements and proportions see Table 1.

Head longer than wide; rostral wider than high; supranasals absent; prefrontals separated from each other by frontal; parietals in contact posteriorly; enlarged nuchal scales in 3 pairs; loreals 2; supraciliaries 7 or 8; supraoculars 4, followed by one small postsupraocular; primary temporal single; secondary temporals 2, upper very large and overlapped by lower one; lower eyelid with a large undivided opaque window, separated from supralabials by a row of small scales; supralabials 7, the fifth and sixth below the eye; ear opening without projecting lobules; tympanum deeply sunk; mental wider than long; infralabials 6; postmental undivided; midbody scales in 28 rows; two medial scale rows on the neck widened; dorsal scales between lateral stripes in ½ + 6 + ½ rows, smooth, larger than lateral scales; paravertebral scales 63–66; ventrals in 61–66 transverse rows, smooth; precloacals 2, enlarged; medial subcaudals widened; limbs short, pentadactyl; fingers and toes meeting when adpressed; subdigital lamellae smooth, 14 under fourth finger and 17–19 under fourth toe.

**Figure 3.**
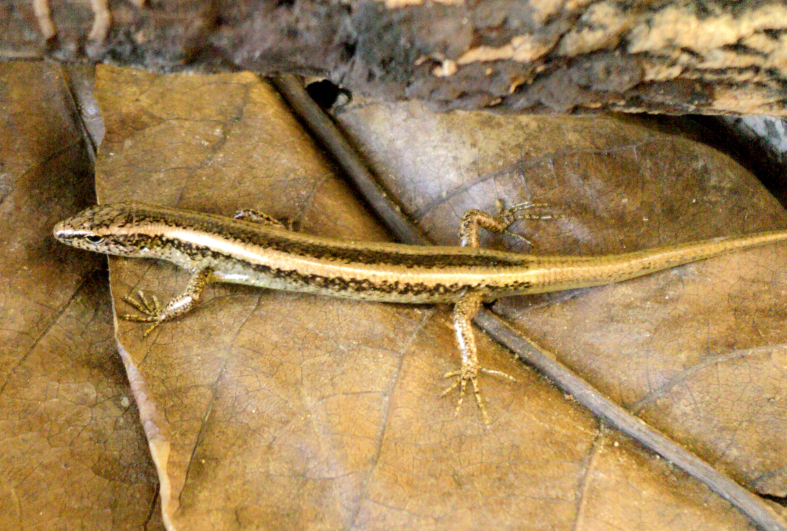
*Scincella devorator* (TBU PAT.72, adult female) from Son La Province, Vietnam

Coloration in alcohol. Dorsum bronze brown, with two silver gray clear bands extending from parietals to base of tail and a dark wide vertebral stripe; numerous blackish spots on the labials; upper lateral zone with a distinct dark stripe from behind the eye to hind limb, with light spots; the lower edge broken in numerous small black dots; venter and under surface of tail base cream. For coloration in life see Fig. 3.

#### Distribution

This species is currently known only from Quang Ninh and Bac Giang provinces in northeastern Vietnam (Nguyen et al., 2009). This is the first record of *Scincella devorator* from northwestern Vietnam.

#### Ecology

The adult females were collected between 10:00 and 16:00 while crossing a forest path. The surrounding habitat was secondary forest of hardwood and shrub.

### Scincella monticola

(Schmidt, 1925)

#### Materials

- **Type status:** Other material. **Occurrence:** catalogNumber: HNUE MNR.56; individualCount: 1; sex: unknown; lifeStage: subadult; **Taxon:** scientificNameID: Scincella monticola; scientificName: Scincella monticola; class: Reptilia; order: Squamata; family: Scincidae; genus: Scincella; specificEpithet: monticola; scientificNameAuthorship: (Schmidt, 1925); **Location:** country: Vietnam; countryCode: VN; stateProvince: Dien Bien; county: Muong Nhe; locality: Muong Nhe Nature Reserve, Sin Thau sector, near Y Ma Ho stream; verbatimElevation: 1742 m; verbatimLatitude: 22°18’34.9''N; verbatimLongitude: 102°10’57''E; verbatimCoordinateSystem: WGS84; **Event:** eventDate: February 18, 2014; eventRemarks: collected by DTL et al.; **Record Level:** language: en; collectionCode: Reptiles; basisOfRecord: PreservedSpecimen

#### Description

Morphological characters (determination after Schmidt 1925, Nguyen et al. 2010c, Nguyen et al. 2010a). Small-sized skink, SVL 27 mm, TaL 37.6 mm. For further measurements and proportions see Table 1.

Head longer than wide; rostral wider than high; supranasals absent; prefrontals separated from each other by frontal; parietals in contact posteriorly; enlarged nuchal scales in 3 pairs; loreals 2; supraciliaries 6; supraoculars 4, followed by a small postsupraocular; primary temporal single; secondary temporals 2, upper very large and overlapped by lower one; lower eyelid with a large undivided opaque window, separated from supralabials by a row of small scales; supralabials 7, the fifth below the eye; ear opening without projecting lobules; tympanum slightly sunk; mental wider than long; infralabials 6; postmental undivided; midbody scales in 24 rows; dorsal scales between lateral stripes in ½ + 4 + ½ rows, smooth, larger than lateral scales; paravertebral scales 58; ventrals in 56 transverse rows, smooth; precloacals 2, enlarged; medial subcaudals widened; limbs short, pentadactyl; fingers and toes widely separated when adpressed; subdigital lamellae smooth, 7 under fourth finger and 11 under fourth toe.

**Figure 4.**
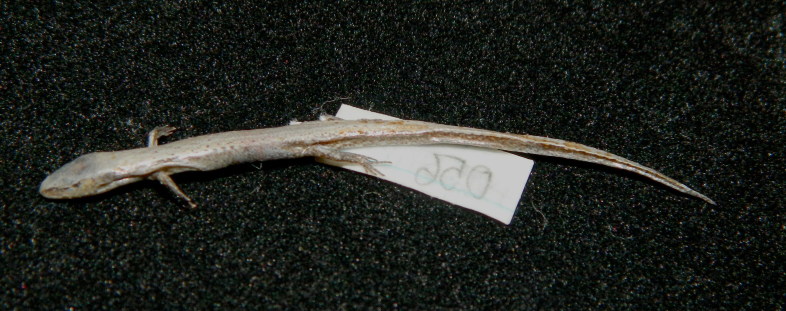
*Scincella monticola* (HNUE MNR.56, subadult in preservative) from Dien Bien Province, Vietnam

Coloration in alcohol. Dorsal surface bronze brown with some indistinct dark spots on body, dark brown laterally with a dark stripe from behind the eye to tail tip; ventral surface cream, underside of tail with some brown spots (see Fig. 4).

#### Distribution

This species was recently recorded in Vietnam from Cao Bang and Lang Son provinces. Elsewhere, this species is known from China (Nguyen et al. 2010 b, c). This is the first record for Dien Bien Province.

#### Ecology

The specimen was collected at ca. 20:00 on the ground, near a stream. The surrounding habitat was evergreen forest of hardwood and shrub.

### Scincella ochracea

(Bourret, 1937)

#### Materials

- **Type status:** Other material. **Occurrence:** catalogNumber: TBU PAT.127; individualCount: 1; sex: male; lifeStage: adult; **Taxon:** scientificNameID: Scincella ochracea; scientificName: Scincella ochracea; class: Reptilia; order: Squamata; family: Scincidae; genus: Scincella; specificEpithet: ochracea; scientificNameAuthorship: (Bourret, 1937); **Location:** country: Vietnam; countryCode: VN; stateProvince: Son La; county: Sop Cop; municipality: Dom Cang; locality: Sop Cop Nature Reserve, near Tin Toc Village; verbatimElevation: 990 m; verbatimLatitude: 20°59’21"N; verbatimLongitude: 103°34’26.6"E; verbatimCoordinateSystem: WGS84; **Event:** eventDate: April 9, 2014; eventRemarks: collected by AVP et al.; **Record Level:** language: en; collectionCode: Reptiles; basisOfRecord: PreservedSpecimen
- **Type status:** Other material. **Occurrence:** catalogNumber: TBU PAT.128; individualCount: 1; sex: male; lifeStage: adult; **Taxon:** scientificNameID: Scincella ochracea; scientificName: Scincella ochracea; class: Reptilia; order: Squamata; family: Scincidae; genus: Scincella; specificEpithet: ochracea; scientificNameAuthorship: (Bourret, 1937); **Location:** country: Vietnam; countryCode: VN; stateProvince: Son La; county: Sop Cop; municipality: Dom Cang; locality: Sop Cop Nature Reserve, near Tin Toc Village; verbatimElevation: 990 m; verbatimLatitude: 20°59’21"N; verbatimLongitude: 103°34’26.6"E; verbatimCoordinateSystem: WGS84; **Event:** eventDate: April 9, 2014; eventRemarks: collected by AVP et al.; **Record Level:** language: en; collectionCode: Reptiles; basisOfRecord: PreservedSpecimen
- **Type status:** Other material. **Occurrence:** catalogNumber: TBU PAT.125; individualCount: 1; sex: female; lifeStage: adult; **Taxon:** scientificNameID: Scincella ochracea; scientificName: Scincella ochracea; class: Reptilia; order: Squamata; family: Scincidae; genus: Scincella; specificEpithet: ochracea; scientificNameAuthorship: (Bourret, 1937); **Location:** country: Vietnam; countryCode: VN; stateProvince: Son La; county: Sop Cop; municipality: Dom Cang; locality: Sop Cop Nature Reserve, near Tin Toc Village; verbatimElevation: 990 m; verbatimLatitude: 20°59’21"N; verbatimLongitude: 103°34’26.6"E; verbatimCoordinateSystem: WGS84; **Event:** eventDate: April 9, 2014; eventRemarks: collected by AVP et al.; **Record Level:** language: en; collectionCode: Reptiles; basisOfRecord: PreservedSpecimen
- **Type status:** Other material. **Occurrence:** catalogNumber: TBU PAT.156; individualCount: 1; sex: male; lifeStage: adult; **Taxon:** scientificNameID: Scincella ochracea; scientificName: Scincella ochracea; class: Reptilia; order: Squamata; family: Scincidae; genus: Scincella; specificEpithet: ochracea; scientificNameAuthorship: (Bourret, 1937); **Location:** country: Vietnam; countryCode: VN; stateProvince: Son La; county: Sop Cop; municipality: Huoi Mot; locality: Sop Cop Nature Reserve, near Pa Man Village; verbatimElevation: 510 m; verbatimLatitude: 21°02’24"N; verbatimLongitude: 103°41’6.6"E; verbatimCoordinateSystem: WGS84; **Event:** eventDate: May 1,2014; eventRemarks: collected by AVP et al.; **Record Level:** language: en; collectionCode: Reptiles; basisOfRecord: PreservedSpecimen
- **Type status:** Other material. **Occurrence:** catalogNumber: TBU PAT.160; individualCount: 1; sex: male; lifeStage: adult; **Taxon:** scientificNameID: Scincella ochracea; scientificName: Scincella ochracea; class: Reptilia; order: Squamata; family: Scincidae; genus: Scincella; specificEpithet: ochracea; scientificNameAuthorship: (Bourret, 1937); **Location:** country: Vietnam; countryCode: VN; stateProvince: Son La; county: Sop Cop; municipality: Huoi Mot; locality: Sop Cop Nature Reserve, near Pa Man Village; verbatimElevation: 510 m; verbatimLatitude: 21°02’24"N; verbatimLongitude: 103°41’6.6"E; verbatimCoordinateSystem: WGS84; **Event:** eventDate: May 1,2014; eventRemarks: collected by AVP et al.; **Record Level:** language: en; collectionCode: Reptiles; basisOfRecord: PreservedSpecimen
- **Type status:** Other material. **Occurrence:** catalogNumber: TBU PAT.155; individualCount: 1; sex: female; lifeStage: adult; **Taxon:** scientificNameID: Scincella ochracea; scientificName: Scincella ochracea; class: Reptilia; order: Squamata; family: Scincidae; genus: Scincella; specificEpithet: ochracea; scientificNameAuthorship: (Bourret, 1937); **Location:** country: Vietnam; countryCode: VN; stateProvince: Son La; county: Sop Cop; municipality: Huoi Mot; locality: Sop Cop Nature Reserve, near Pa Man Village; verbatimElevation: 510 m; verbatimLatitude: 21°02’24"N; verbatimLongitude: 103°41’6.6"E; verbatimCoordinateSystem: WGS84; **Event:** eventDate: May 1,2014; eventRemarks: collected by AVP et al.; **Record Level:** language: en; collectionCode: Reptiles; basisOfRecord: PreservedSpecimen
- **Type status:** Other material. **Occurrence:** catalogNumber: TBU PAT.157; individualCount: 1; sex: female; lifeStage: adult; **Taxon:** scientificNameID: Scincella ochracea; scientificName: Scincella ochracea; class: Reptilia; order: Squamata; family: Scincidae; genus: Scincella; specificEpithet: ochracea; scientificNameAuthorship: (Bourret, 1937); **Location:** country: Vietnam; countryCode: VN; stateProvince: Son La; county: Sop Cop; municipality: Huoi Mot; locality: Sop Cop Nature Reserve, near Pa Man Village; verbatimElevation: 510 m; verbatimLatitude: 21°02’24"N; verbatimLongitude: 103°41’6.6"E; verbatimCoordinateSystem: WGS84; **Event:** eventDate: May 1,2014; eventRemarks: collected by AVP et al.; **Record Level:** language: en; collectionCode: Reptiles; basisOfRecord: PreservedSpecimen
- **Type status:** Other material. **Occurrence:** catalogNumber: TBU PAT.159; individualCount: 1; sex: female; lifeStage: adult; **Taxon:** scientificNameID: Scincella ochracea; scientificName: Scincella ochracea; class: Reptilia; order: Squamata; family: Scincidae; genus: Scincella; specificEpithet: ochracea; scientificNameAuthorship: (Bourret, 1937); **Location:** country: Vietnam; countryCode: VN; stateProvince: Son La; county: Sop Cop; municipality: Huoi Mot; locality: Sop Cop Nature Reserve, near Pa Man Village; verbatimElevation: 510 m; verbatimLatitude: 21°02’24"N; verbatimLongitude: 103°41’6.6"E; verbatimCoordinateSystem: WGS84; **Event:** eventDate: May 1,2014; eventRemarks: collected by AVP et al.; **Record Level:** language: en; collectionCode: Reptiles; basisOfRecord: PreservedSpecimen
- **Type status:** Other material. **Occurrence:** catalogNumber: TBU PAT.161; individualCount: 1; sex: female; lifeStage: adult; **Taxon:** scientificNameID: Scincella ochracea; scientificName: Scincella ochracea; class: Reptilia; order: Squamata; family: Scincidae; genus: Scincella; specificEpithet: ochracea; scientificNameAuthorship: (Bourret, 1937); **Location:** country: Vietnam; countryCode: VN; stateProvince: Son La; county: Sop Cop; municipality: Huoi Mot; locality: Sop Cop Nature Reserve, near Pa Man Village; verbatimElevation: 510 m; verbatimLatitude: 21°02’24"N; verbatimLongitude: 103°41’6.6"E; verbatimCoordinateSystem: WGS84; **Event:** eventDate: May 1,2014; eventRemarks: collected by AVP et al.; **Record Level:** language: en; collectionCode: Reptiles; basisOfRecord: PreservedSpecimen
- **Type status:** Other material. **Occurrence:** catalogNumber: IEBR A.2014.23; individualCount: 1; sex: male; lifeStage: adult; **Taxon:** scientificNameID: Scincella ochracea; scientificName: Scincella ochracea; class: Reptilia; order: Squamata; family: Scincidae; genus: Scincella; specificEpithet: ochracea; scientificNameAuthorship: (Bourret, 1937); **Location:** country: Vietnam; countryCode: VN; stateProvince: Son La; county: Sop Cop; municipality: Pung Banh; locality: Sop Cop Nature Reserve, near Kha Village; verbatimElevation: 1230 m; verbatimLatitude: 21°00’20.5"N; verbatimLongitude: 103°20’17.6E; verbatimCoordinateSystem: WGS84; **Event:** eventDate: September 20, 2014; eventRemarks: collected by TQN et al.; **Record Level:** language: en; collectionCode: Reptiles; basisOfRecord: PreservedSpecimen
- **Type status:** Other material. **Occurrence:** catalogNumber: HNUE MNR.88; individualCount: 1; sex: male; lifeStage: adult; **Taxon:** scientificNameID: Scincella ochracea; scientificName: Scincella ochracea; class: Reptilia; order: Squamata; family: Scincidae; genus: Scincella; specificEpithet: ochracea; scientificNameAuthorship: (Bourret, 1937); **Location:** country: Vietnam; countryCode: VN; stateProvince: Dien Bien; county: Muong Nhe; locality: Muong Nhe Nature Reserve, Chung Chai sector, near Doan Ket Village; verbatimElevation: 592 m; verbatimLatitude: 22°18’25"N; verbatimLongitude: 102°23’42"E; verbatimCoordinateSystem: WGS84; **Event:** eventDate: August 5, 2014; eventRemarks: collected by DTL et al.; **Record Level:** language: en; collectionCode: Reptiles; basisOfRecord: PreservedSpecimen

#### Description

Morphological characters (determination after Bourret 1937, Ouboter 1986). Males: SVL 34.2–45.4 mm (mean ± SD 44 ±1.4 mm, n = 5), TaL 61.8–75.0 mm (n = 2); females: SVL 43.2–50.0 mm (mean ± SD 46.5±2.5 mm, n = 5), TaL 63.9 mm (n = 1). For further measurements and proportions see Table 1.

Head longer than wide; rostral wider than high; supranasals absent; prefrontals in contact with each other; parietals in contact posteriorly; enlarged nuchal scales in 0–3 pairs; loreals 2; supraciliaries 7 or 8; supraoculars 4, followed by two small scales; primary temporals 2; secondary temporals 2, upper very large and overlapped by lower one; lower eyelid with a large undivided opaque window, separated from supralabials by 1–3 row of small scales; supralabials 7 (rarely 8), the fifth and sixth below the eye; ear opening with 3 or 4 projecting lobules; tympanum deeply sunk; mental wider than long; infralabials 6 (rarely 5); postmental undivided; midbody scales in 30 or 32 rows; dorsal scales between lateral stripes in ½ + 6 + ½ rows, smooth, as large as lateral scales; paravertebral scales 61–67; ventrals in 66–71 transverse rows, smooth; precloacals 2, enlarged; medial subcaudals slightly widened; limbs short, pentadactyl; fingers and toes widely separated when adpressed; subdigital lamellae smooth, 9–11 under fourth finger and 15–17 under fourth toe.

**Figure 5.**
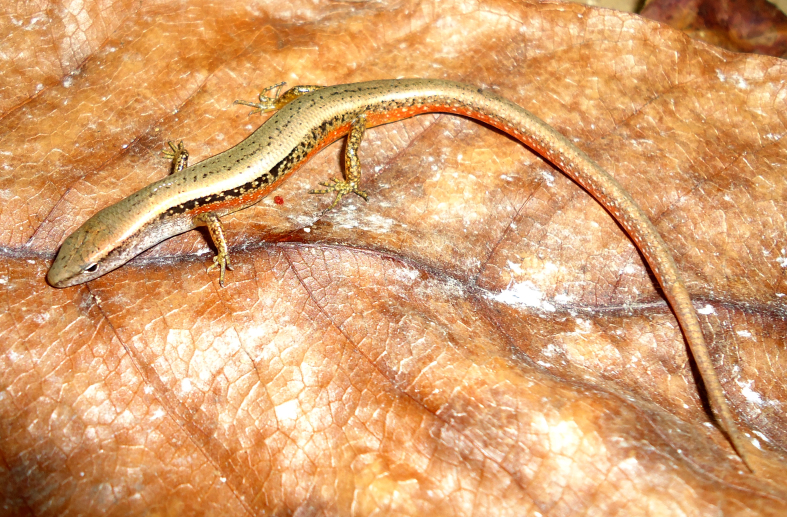
*Scincella ochracea* (TBU PAT.156, adult male) from Son La Province, Vietnam.

Coloration in alcohol. Dorsum silver gray, with a dark vertebral stripe; upper lateral zone with a distinct reddish band from behind the eye to hind limb, with some light spots; the lower edge broken in somes small black dots; venter and underside of tail base cream or whitish. For coloration in life see Fig. 5.

#### Distribution

In Vietnam, this species has been recorded from Lai Chau Province (Eremchenko, 2003). Elsewhere, this species is known from Laos (Bourret, 1937). This is the first record of the species from Son La and Dien Bien provinces.

#### Ecology

Specimens were collected between 10:00 and 16:30 on the ground. The surrounding habitat was grass and shrub near a forest path.

### Sphenomorphus cryptotis

Darevsky, Orlov & Ho, 2004

#### Materials

- **Type status:** Other material. **Occurrence:** catalogNumber: HNUE MNR.26; individualCount: 1; sex: female; lifeStage: adult; **Taxon:** scientificNameID: Sphenomorphus cryptotis; scientificName: Sphenomorphus cryptotis; class: Reptilia; order: Squamata; family: Scincidae; genus: Sphenomorphus; specificEpithet: cryptotis; scientificNameAuthorship: Darevsky, Orlov & Ho, 2004; **Location:** country: Vietnam; countryCode: VN; stateProvince: Dien Bien; county: Muong Nhe; locality: Muong Nhe Nature Reserve, Sin Thau sector, Y Ma Ho stream; verbatimElevation: 890 m; verbatimLatitude: 22°14’15"N; verbatimLongitude: 103°25’9.6"E; verbatimCoordinateSystem: WGS84; **Event:** eventDate: February 18, 2013; eventRemarks: collected by DTL et al.; **Record Level:** language: en; collectionCode: Reptiles; basisOfRecord: PreservedSpecimen

#### Description

Morphological characters (determination after Darevsky et al. 2004). Female: SVL 68.2 mm; TaL 116.8 mm. For further measurements and proportions see Table 1.

Head longer than wide; rostral wider than high; supranasals absent; prefrontals in contact with each other; parietals in contact posteriorly; loreals 2; supraoculars 4, followed by 2 postsupraoculars, anterior one divided; primary temporal single; secondary temporals 2, upper very large and overlapped by lower one; lower eyelid scaly; supralabials 7, the fifth and sixth below the eye, separated from the eye by one row of small scales; external ear openings superficial, without lobules; mental wider than long; infralabials 6; postmental undivided; midbody scales in 34 rows; dorsal scales between lateral stripes in ½ + 6 + ½ rows, smooth; paravertebral scales 71; ventrals in 75 transverse rows, smooth; precloacals 2, enlarged; medial subcaudals widened; limbs short, pentadactyl; fingers and toes meeting when adpressed; subdigital lamellae smooth, numbering 13 under fourth finger and 20 under fourth toe.

**Figure 6.**
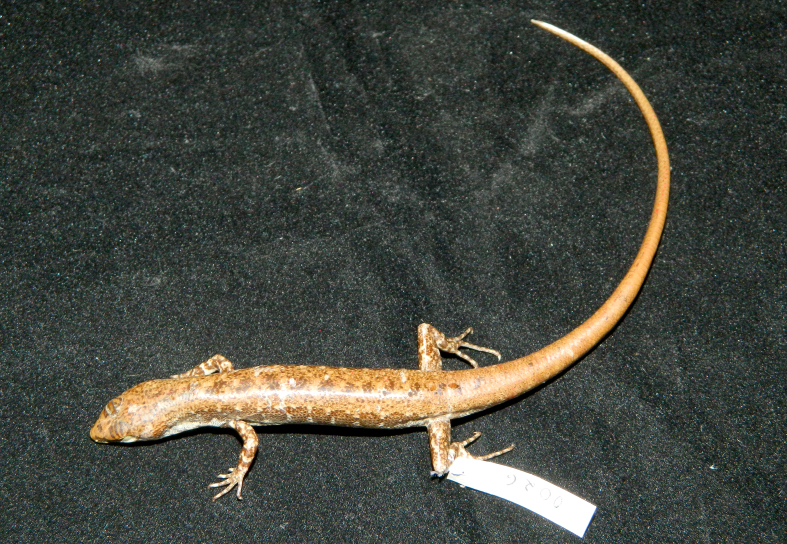
*Sphenomorphus cryptotis* (HNUE MNR.26, adult female in preservative) from Dien Bien Province, Vietnam.

Coloration in alcohol. Dorsum and tail base yellowish brown with a vertebral row of large black spots; numerous indistinct white spots on the labials; lateral zone with a distinct dark stripe from behind the eye to tail base, with white spots; neck and throat white, with black dots; venter and underside anterior part of tail white, posterior part of tail yellowish brown (see Fig. 6).

#### Distribution

This species is currently known only from northern Vietnam: Lao Cai, Bac Giang, Quang Ninh, and Nghe An provinces (Nguyen et al., 2009). This is the first record of the species from Dien Bien Province.

#### Ecology

The adult female was collected at ca. 9:00 on a tree branch, ca. 1.2 m above the water in a rocky stream. The surrounding habitat was evergreen secondary forest of hardwood and shrub.

### Sphenomorphus indicus

(Gray, 1853)

#### Materials

- **Type status:** Other material. **Occurrence:** catalogNumber: HNUE MNR.20; individualCount: 1; sex: female; lifeStage: adult; **Taxon:** scientificNameID: Sphenomorphus indicus; scientificName: Sphenomorphus indicus; class: Reptilia; order: Squamata; family: Scincidae; genus: Sphenomorphus; specificEpithet: indicus; scientificNameAuthorship: (Gray, 1853); **Location:** country: Vietnam; countryCode: VN; stateProvince: Dien Bien; county: Muong Nhe; locality: Muong Nhe Nature Reserve, Sin Thau sector; verbatimElevation: 1742 m; verbatimLatitude: 22°18’34.9"N; verbatimLongitude: 102°10’57"E; verbatimCoordinateSystem: WGS84; **Event:** eventDate: February 17, 2013; eventRemarks: collected by DTL et al.; **Record Level:** language: en; collectionCode: Reptiles; basisOfRecord: PreservedSpecimen
- **Type status:** Other material. **Occurrence:** catalogNumber: HNUE MNR.27; individualCount: 1; sex: male; lifeStage: adult; **Taxon:** scientificNameID: Sphenomorphus indicus; scientificName: Sphenomorphus indicus; class: Reptilia; order: Squamata; family: Scincidae; genus: Sphenomorphus; specificEpithet: indicus; scientificNameAuthorship: (Gray, 1853); **Location:** country: Vietnam; countryCode: VN; stateProvince: Dien Bien; county: Muong Nhe; locality: Muong Nhe Nature Reserve, Sin Thau sector; verbatimElevation: 1742 m; verbatimLatitude: 22°18’34.9"N; verbatimLongitude: 102°10’57"E; verbatimCoordinateSystem: WGS84; **Event:** eventDate: February 17, 2013; eventRemarks: collected by DTL et al.; **Record Level:** language: en; collectionCode: Reptiles; basisOfRecord: PreservedSpecimen
- **Type status:** Other material. **Occurrence:** catalogNumber: HNUE MNR.28; individualCount: 1; sex: male; lifeStage: adult; **Taxon:** scientificNameID: Sphenomorphus indicus; scientificName: Sphenomorphus indicus; class: Reptilia; order: Squamata; family: Scincidae; genus: Sphenomorphus; specificEpithet: indicus; scientificNameAuthorship: (Gray, 1853); **Location:** country: Vietnam; countryCode: VN; stateProvince: Dien Bien; county: Muong Nhe; locality: Muong Nhe Nature Reserve, Sin Thau sector; verbatimElevation: 1742 m; verbatimLatitude: 22°18’34.9"N; verbatimLongitude: 102°10’57"E; verbatimCoordinateSystem: WGS84; **Event:** eventDate: February 17, 2013; eventRemarks: collected by DTL et al.; **Record Level:** language: en; collectionCode: Reptiles; basisOfRecord: PreservedSpecimen
- **Type status:** Other material. **Occurrence:** catalogNumber: HNUE MNR.65; individualCount: 1; sex: female; lifeStage: adult; **Taxon:** scientificNameID: Sphenomorphus indicus; scientificName: Sphenomorphus indicus; class: Reptilia; order: Squamata; family: Scincidae; genus: Sphenomorphus; specificEpithet: indicus; scientificNameAuthorship: (Gray, 1853); **Location:** country: Vietnam; countryCode: VN; stateProvince: Dien Bien; county: Muong Nhe; locality: Muong Nhe Nature Reserve, Chung Chai sector; verbatimElevation: 848 m; verbatimLatitude: 22°13’57"N; verbatimLongitude: 102°22’49.9"E; verbatimCoordinateSystem: WGS84; **Event:** eventDate: March 21, 2014; eventRemarks: collected by DTL et al.; **Record Level:** language: en; collectionCode: Reptiles; basisOfRecord: PreservedSpecimen

#### Description

Morphological characters (determination after Smith 1935, Taylor 1963, Nguyen et al. 2011). Large-sized skinks, females: SVL 78.8–82.6 mm (n = 2), TaL 141.3–151 mm (n = 2); males: SVL 63.4–69.6 mm (n = 2), TaL 112.2 mm (n = 1). For further measurements and proportions see Table 1.

Head longer than wide; rostral wider than high; supranasals absent; prefrontals separated from each other by frontal; parietals in contact posteriorly; one pair of enlarged nuchal scales; loreals 2; supraoculars 4, followed by two small postsupraoculars; primary temporals 2; secondary temporals 2; lower eyelid scaly; supralabials 7, the fifth and sixth below the eye, separated from it by a row of small scales; external ear present, with three very small lobules, tympanum deeply sunk; mental wider than long; infralabials 7; postmental undivided; midbody scales in 34–36 rows; dorsal scales between lateral stripes in ½ + 8 + ½ rows, smooth, as large as the lateral scales; paravertebral scales 68–74; ventrals in 65–69 transverse rows, smooth; precloacals 2, enlarged; medial subcaudals not widened; limbs short, pentadactyl; fingers and toes meeting when adpressed; subdigital lamellae smooth, 11 under fourth finger and 15–18 under fourth toe.

**Figure 7.**
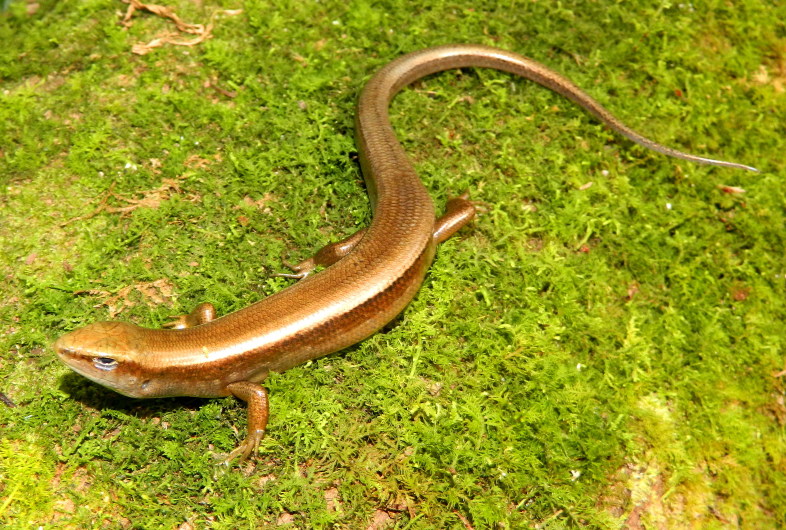
*Sphenomorphus indicus* (HNUE MNR.65, adult female) from Dien Bien Province, Vietnam

Coloration in alcohol. Dorsum and tail base bronze brown; upper lateral zone with a dark gray stripe, in width of 3 or 4 scales, from behind eye to tail base; light dorsolateral stripe present on neck and shoulder, the lower margin of dark stripe with some broken light spots; lower lateral zone light gray; ventral white. For coloration in life see Fig. 7.

#### Distribution

In Vietnam, this species is a common species known from Lao Cai and Lang Son provinces in the North southwards to Dong Nai Province. Elsewhere, this species has been recorded from India, Bhutan, China, Taiwan, Myanmar, Laos, Thailand, Cambodia, Malaysia, and Indonesia (Nguyen et al., 2009). This is the first record of the species in Dien Bien Province.

#### Ecology

The specimens were collected between 9:00 and 12:00 on the ground. The surrounding habitat was secondary forest of small hardwood, bamboo and shrub.

## Discussion

Except for *Eutropis macularius* and *Sphenomorphus indicus*, the remaining recorded species from Dien Bien and Son La provinces are poorly known in Vietnam due to their rarity or cryptic lifestyle in or under the leaf litter (Darevsky et al. 2004, Nguyen et al. 2010c, Nguyen et al. 2010a). *Scincella devorator* and *Sphenomorphus cryptotis* were recently described from northern Vietnam by Darevsky et al. (2004) based on a collection from Nghe An and Quang Ninh provinces. *Scincella ochracea* and *S. monticola* were recorded for the first time from the country by Eremchenko (2003) and by Nguyen et al. (2010c), respectively. Our new findings increase the total number of the family Scincidae species to nine in Dien Bien Province and 14 in Son La Province, comprising five species of *Eutropis*, one species of *Lygosoma*, two species of *Plestiodon*, five species of *Scincella*, two species of *Sphenomorphus*, and four species of *Tropidophorus* (see Table 2).

## Supplementary Material

Supplementary material 1 Appendix 1. Morphological characters of skink specimens from Vietnam (for abbreviations see Material and Methods; * regenerated tail or tai tip lost; – data unobtainable; bilateral scale counts are given as left/right) Data type: Morphological characters
File: oo_37666.xlsx Pham, Le, Nguyen, Ziegler and Nguyen

XML Treatment for Eutropis macularius

XML Treatment for Scincella devorator

XML Treatment for Scincella monticola

XML Treatment for Scincella ochracea

XML Treatment for Sphenomorphus cryptotis

XML Treatment for Sphenomorphus indicus

## Figures and Tables

**Table 1.** Measurements (in mm) and proportions of skink specimens from northwestern Vietnam.

Cha-racter	*Eutropis macularius*				*Scincella devorator*	*Scincella monticola*	*Scincella ochracea*				*Sphenomorphus cryptotis*	*Sphenomorphus indicus*	
	Min– Max (n=5 ♂)	Mean± SD ♂)	Min–Max ♀)	Mean± SD (n=4 ♀)	Min– Max (n=2 ♀)	(n=1 ♀)	Min– Max (n=6 ♂)	Mean± SD (n=6 ♂)	Min– Max (n=5 ♀)	Mean± SD (n=5 ♀)	(n=1 ♀)	Min–Max (n=2 ♂)	Min–Max (n=2 ♀)
SVL	55.6–61.7	58.3± 2.4	55.4–59.6	57.5± 1.7	52.1–53.9	27.0	34.2–45.4	42.0± 4.6	43.2–50.0	46.5± 2.5	68.2	63.4–69.6	78.8–82.6
TaL	95.4	95.4	80.5–85.7	83.6± 2.7	–	37.6	62.3–75.0	68.82± 4.2	63.9	–	116.8	112.2	141.3–151.0
AG	24.1–30.4	27.2± 2.9	26.7–29.0	27.5± 1.0	27.3– 28	13.8	17.9–24.4	21.5± 2.4	26.1–30.0	27.4± 1.5	32.8	32.7–30.1	40.6–41.0
SL	4.8–5.6	5.2± 0.3	5.0– 5.2	5.0± 0.1	4.3– 4.4	2.4	3.0–3.4	3.2± 0.1	2.4–3.0	2.8± 0.3	6.4	5.5–5.3	6.9
STL	11.6–13.0	12.4± 0.5	11.2–12.0	11.6± 0.3	9.9– 10.1	5.5	6.8–8.7	8.0± 0.7	7.0–7.9	7.6± 0.4	14.6	12.6–12.8	16.5–16.6
SFlL	21.1–24.0	22.1± 1.2	19.5–22.2	20.6± 1.2	18.0–18.8	10.4	12.9–16.2	15.3± 1.4	12.4–15.9	14.5± 1.4	27.3	21.9	29.8–30.7
END	3.2–3.5	3.3± 0.1	3.0– 3.8	3.5± 0.3	2.9	1.1	1.8–2.1	1.9± 0.2	1.8–2.2	2.0± 0.2	4.1	3.5–3.7	4.3– 4.5
EL	3.4–4.0	3.7± 0.2	3.0– 3.8	3.4± 0.3	1.1– 1.5	1.6	1.8–2.9	2.3± 0.5	1.7–2.7	2.1± 0.4	4.6	3.9	4.1– 4.4
HL	10.6–11.1	10.9± 0.2	9.3– 9.7	9.6± 0.2	9.4– 9.5	5.6	6.4–8.2	7.9± 0.7	6.9–8.0	7.5± 0.5	14.3	11.6–11.9	14.3–14.6
HW	7.6–9.3	8.7± 0.7	8.1– 8.9	8.6± 0.4	7.2– 7.1	4.0	4.5–5.8	5.3± 0.5	4.8–10.4	6.1± 2.4	9.9	9.0–9.9	11.9–12.2
HH	6.4–7.5	7.0± 0.4	6.7– 7.3	7.0± 0.3	5.4– 5.6	2.8	3.4–4.4	4.10± 0.4	3.6–4.3	3.9± 0.3	7.8	6.7–7.0	9.7– 9.8
TYD	1.3–1.4	1.3± 0.1	1.1– 1.4	1.3± 0.1	1.0– 1.1	1.0	1.2–1.5	1.4± 0.1	1.3–1.8	1.4± 0.2	1.5	2.1–2.2	2.4– 2.6
FlL	17.2–20.2	18.3± 1.3	16.8–17.8	17.4± 0.4	17.0–17.2	5.8	9.2–10.4	9.8± 0.5	7.6–9.7	8.9± 0.8	19.3	17.5–18.6	20.0–20.6
HlL	25.9–27.9	26.4± 1.0	24.7–25.0	24.8± 0.2	21.6–22.1	7.8	12.7–16.6	15.0± 1.4	13.9–15.2	14.6± 0.5	28.2	26.6–27.6	30.7–34.9
SVL/ TaL	0.65	0.65	0.65–0.74	0.69± 0.05	–	0.72	0.58–0.72	0.65± 0.1	0.72	–	0.6	0.62	0.52–0.58
FIL/ SVL	0.31–0.33	0.32± 0.01	0.29–0.31	0.3± 0.01	0.32–0.33	0.21	0.22–0.27	0.24± 0.02	0.17–0.21	0.2± 0.01	0.28	0.25–0.29	0.25
HIL/ SVL	0.45–0.47	0.46± 0.01	0.41–0.45	0.43± 0.02	0.4– 0.42	0.29	0.34–0.38	0.36± 0.02	0.3–0.32	0.3± 0.01	0.41	0.38–0.44	0.39–0.42

**Table 2.** Checklist of skinks (Scincidae) recorded from Dien Bien and Son La provinces (after Bobrov and Ho 1993, Do and Le 2009, Nguyen et al. 2009, Nguyen et al. 2010, Nguyen et al. 2010a, * = new provicial record)

Name	Dien Bien	Son La
*Eutropis chapaensis* (Bourret, 1937)		x
*Eutropis darevskii* (Bobrov, 1992)		x
*Eutropis longicaudatus* (Hallowell, 1856)	x	x
*Eutropis macularius* (Blyth, 1853)*		x
*Eutropis multifasciatus* (Kuhl, 1820)	x	x
*Lygosoma quadrupes* (Linnaeus, 1766)	x	
*Plestiodon quadrilineatus* Blyth, 1853		x
*Plestiodon tamdaoensis* (Bourret, 1937)		x
*Scincella darevskii* Nguyen, Ananjeva, Orlov, Rybaltovsky & Böhme, 2010	x	
*Scincella devorator* (Darevsky, Orlov & Ho, 2004)*		x
*Scincella monticola* (Schmidt, 1925)*	x	
*Scincella reevesii* (Gray, 1838)		x
*Scincella ochracea* (Bourret, 1937)*	x	x
*Sphenomorphus cryptotis* Darevsky, Orlov & Ho, 2004*	x	
*Sphenomorphus indicus* (Gray, 1853)*	x	x
*Tropidophorus baviensis* Bourret, 1939		x
*Tropidophorus berdmorei* (Blyth,1853)		x
*Tropidophorus hainanus* Smith, 1923	x	
*Tropidophorus microlepis* Günther, 1861		x
